# Supplementary material for: Emergence of global synchronization in directed excitatory networks of type I neurons
Source: Sci Rep. 2020 Feb 24;10:3306. doi: 10.1038/s41598-020-60205-0 (PMC7039997; doi:10.1038/s41598-020-60205-0)
Supplement: Supplementary file 1 — Supplementary Information. [file 41598_2020_60205_MOESM1_ESM.pdf]

Supplementary Material

Abolfazl Ziaeemehr[1], Mina Zarei[1,2], and Aida Sheshbolouki[3]

[1]Institute of Advanced Studies in Basic Sciences (IASBS),Department of Physics, Zanjan, 45137-66731, Iran.
[2]Institute for Research in Fundamental Sciences (IPM), School of Computer Science, Tehran, 19395-5531, Iran
[3]University of Waterloo, Cheriton School of Computer Science, Waterloo, N2L3G1, Canada

Phase model

Analytical study of the stability of the synchronized motifs

In this section, we study the stability of the feedback and feedforward loops constructed from the identical type I or type II phase oscillators.

We consider the reduced two-dimensional systems of the phase differences. Therefore, we define $\omega_i = \omega$, $\theta_1 - \theta_2 = \phi_1$, $\theta_2 - \theta_3 = \phi_2$, $\theta_1 - \theta_3 = \phi_1 + \phi_2$. Two phases $\theta_1(t)$ and $\theta_2(t)$ are synchronized if their difference $\phi_1(t)$ is bounded at the stationary state.

Using equation 1 (from the main text) and considering **the type II phase oscillators** ($u_i = 1$) **situated in a FBL**, the phase differences $\phi_1(t)$ and $\phi_2(t)$ clearly satisfy the following equations:

$$\begin{aligned} \dot{\phi_1} &= -\sigma\left[\sin(\phi_1+\phi_2)+\sin(\phi_1)\right] \ , \\ \dot{\phi_2} &= \sigma\left[\sin(\phi_1)-\sin(\phi_2)\right] \ , \end{aligned} \tag{1}$$

This system has different fixed points $(\phi_1^*, \phi_2^*) = (0,0), (0,\pm\pi), (\pm\pi, 0), (\pm\frac{2\pi}{3}, \pm\frac{2\pi}{3})$. Where, the synchronous state $(\phi_1^*, \phi_2^*) = (0,0)$ is stable (unstable) for excitatory (inhibitory having negative σ) phase oscillators.

In the case of the **identical type II FFLs**, the evolutions of the phase differences are given by the following equations:

$$\begin{aligned} \dot{\phi_1} &= -\sigma\sin(\phi_1) \ , \\ \dot{\phi_2} &= \sigma\left[\sin(\phi_1)-\sin(\phi_2)-\sin(\phi_1+\phi_2)\right] \ , \end{aligned} \tag{2}$$

The system has one stable (unstable) fixed point ($(\phi_1^*, \phi_2^*) = (0,0)$) for excitatory (inhibitory) oscillators, and two lines of unstable fixed points $(\pm\pi, \phi_2^*)$ for both exci-

tatory and inhibitory oscillators. Therefore, according to the above equations identical excitatory type II motifs are synchronized, but not the inhibitory ones.

For the **identical type I FBLs**, the reduced two-dimensional system is as follows:

$$\begin{aligned}\dot{\phi}_1 &= \frac{\sigma}{2}\left[\cos(\phi_1) - \cos(\phi_1 + \phi_2)\right] \ , \\ \dot{\phi}_2 &= \frac{\sigma}{2}\left[\cos(\phi_2) - \cos(\phi_1)\right] \ , \end{aligned} \tag{3}$$

This system has three fixed points: $(\phi_1^*, \phi_2^*) = (0,0), (\pm\frac{2\pi}{3}, \pm\frac{2\pi}{3})$. In the cases of $((\phi_1^*, \phi_2^*) = (\pm\frac{2\pi}{3}, \pm\frac{2\pi}{3}))$, the phases of oscillators are located at three equidistant points around the unit circle (i.e. asynchronous state). In fact, asynchronous states are stable for excitatory and inhibitory type I FBLs.

In the case of the **identical type I FFLs**, the evolutions of the phase differences are given as follows:

$$\begin{aligned}\dot{\phi}_1 &= -\frac{\sigma}{2}(1 - \cos(\phi_1)) \ , \\ \dot{\phi}_2 &= \frac{\sigma}{2}\left[\cos(\phi_2) - \cos(\phi_1) + \cos(\phi_1 + \phi_2) - 1\right] \ , \end{aligned} \tag{4}$$

This system has just one fixed point at $(\phi_1^*, \phi_2^*) = (0,0)$. At this fixed point, both eigenvalues of the Jacobian matrix are zero. Therefore, this fixed point is globally stable, but not asymptotically stable. In fact, if one perturbs the system and then leaves the system alone, the time dependence of the perturbation would be milder than exponential. Therefore, identical type I excitatory and inhibitory FFLs are synchronized. The phase plane portraits have been depicted on the **figure 1** of the manuscript.

Phase response curve of a phase oscillator

The phase response curve of a phase oscillator modeled by the generalized Kuramoto model is given by $G(\theta) = u\sin(\theta) + (1-u)\frac{(1-cos(\theta))}{2}$. Where $u = 0$ and $u = 1$ correspond to the type I and type II oscillators, respectively (see **figure S1**).

Calculation of the Lyapunov exponents

Considering our continuous dynamical system in an n-dimensional phase space, we monitor the long-term evolution of an infinitesimal n-ellipsoid of initial conditions. The *i*th one-dimensional Lyapunov exponent is then defined in terms of the length of the ellipsoidal principal axis $p_i(t)$ [1]:

$$\lambda_i = \lim_{t\to\infty} \frac{1}{t} \log_2 \frac{p_i(t)}{p_i(0)}, \tag{5}$$

where the Lyapunov exponents will be arranged such that: $\lambda_1 \geq \lambda_2 \geq \ldots \geq \lambda_n$. The λ_1 and λ_n correspond to the most rapidly expanding and contracting principal axes, respectively. We simultaneously solve *n* equations of the system and $n \times n$ equations of the linearized system. The growth of the corresponding set of vectors is measured,

and as the system evolves, the vectors are repeatedly reorthonormalized (e.g. every 50 time steps) using the Gram-Schmidt Orthogonalization (GSO). This makes the vectors maintain a proper phase space orientation.

The simulation programs were written in C++ and using Runge Kutta 4th order integration scheme from Boost odeint library and time step of 0.01 time unit.

The steady-state is determined by convergence test. The simulation program memorizes subsequent values of each lyapunov exponent λ_i in *N* buffers of a fixed capacity (e.g 2500 points). When the buffers are full, the program calculates standard deviation of all the values in each of *N* buffers. If the standard deviation of values in any buffer is too high (compared to a fixed threshold e.g. 10^{-6}), then the buffers are cleared and computations are continued. Otherwise, if the value of standard deviation for each buffer is below a fixed threshold, the calculations are terminated. The final value of λ_i returned by the program is equal to the average of all the values λ_i memorized in the *i*th buffer [2].

The Lyapunov exponents versus time for identical type I and type II excitatory phase oscillators connected by different scale-free networks are depicted in the **Figure S2**.

Synchronization of the inhibitory phase oscillators

Figure S3 compares the synchronizability of the excitatory and the inhibitory scale-free undirected and oriented graphs. We can see that only the DAGs constructed from type I inhibitory oscillators are synchronized.

Gradually adding feedback loops to the DAGs

The effect of gradually adding feedback loops to the DAGs is investigated in the **figure S4**. The loops are added randomly to the DAGs and their number is calculated by Trace$(A^3)/3$. We can see that the synchronizability of the scale-free directed networks decreases nonlinearly with increasing the number of feedback loops. The high standard deviations indicate the fact that in addition to the number of the feedback loops, their positions are also important on the network synchronization.

Merged inhibitory and excitatory feedforward motifs

We show that type I inhibitory and excitatory connected DAGs (i.e. the networks that have a single source node from which all other nodes are reachable) are synchronized. However, in reality inhibitory and excitatory neurons work together to perform complex tasks. To address this issue, we consider the aggregation of two feedforward motifs that their effective nodes (i.e. nodes which have outgoing edges) are purely inhibitory or excitatory (see **Figure S5**). The results show that, type I oscillators (but not the type II ones) are synchronized when these motifs are merged together and form a larger connected network. On the other hand, when the motifs joined to each other and generate a disconnected network, the global synchronization of the oscillators breaks, as one would expect. The results we derive are not changed qualitatively by swapping

excitatory and inhibitory nodes. In general, excitatory and inhibitory inputs of a neuron are said to be balanced, and this balance is important for the highly irregular firing observed in the cortex. It seems that, disconnected networks constructed from motifs with pure effective nodes can provide these kinds of patterns. Note that, the global dynamics of motifs with hybrid effective nodes is more complex and directly related to the initial phase values. Therefore, further investigations are required to understand the relationship between structure and dynamics of large hybrid networks.

Neuron model

Characteristics of the neuron models

Tables S1 and **S2** summarize the values we used in our simulations for the parameters of the Wang-Buzsáki and Traub models. The characteristics of Wang-Buzsáki and Traub type I neurons are presented in the **figure S6** and **figure S8**, respectively. As we expected, the frequency-current curve for type I neurons is continuous and the neuron fires at arbitrarily low frequencies. In addition, the phase response curve is an exclusively positive curve.

Voltage synchrony measure

We also used the voltage synchrony measure to monitor the degree of spike synchrony in the networks. This measure provides evaluations of long-term fluctuations in the global potential as described by the following formula:

$$M = \frac{\sqrt{\langle V_g(t)^2\rangle_t - \langle V_g(t)\rangle_t^2}}{\frac{1}{N}\sum_{i=1}^{N}\sqrt{\langle V_i(t)^2\rangle_t - \langle V_i(t)\rangle_t^2}}, \quad V_g(t) = \frac{1}{N}\sum_{i=1}^{N} V_i(t).$$

where N is the number of neurons in the network, and $V_g(t)$ is the average membrane potentials of the neurons in the population (global potential). Here, $\langle \dots \rangle_t$ denotes time-averaging over a large time interval. To normalize the measure, this value is divided by the average fluctuations in the membrane potentials of single neurons. The value of M is bounded between 0 and 1, where $M = 1$ and $M = 0$ indicate fully synchronized and asynchronous states, respectively.

The synchronizations of type I Wang-Buzsáki inhibitory and Traub excitatory neurons connected by scale-free DAGs have been also investigated using voltage synchrony measure (see **figure S9** and **figure S10**). The results are similar to that of found using interspike distance synchrony measure (see **figures 6** and **7** in the manuscript). It means that there exist parameter regions in which type I inhibitory and excitatory neurons are synchronized on scale-free DAGs.

The effects of parameter ϕ of the Wang-Buzsáki model

The parameter ϕ in the Wang-Buzsáki mode affects Neuronal afterhyperpolarizations. Decreasing ϕ means reducing α_h, β_h, α_n, and β_n, that is to say slowing down h and

Table S1: Parameters for Wang-Buzsáki neurons.

parameter	value
g_L	0.1 (mS/cm^2)
g_{Na}	35 (mS/cm^2)
g_K	9 (mS/cm^2)
E_L	-65 (mV)
E_{Na}	55 (mV)
E_K	-90 (mV)
E_{syn}	-75 (mV)
g_{syn}	0.1 (mS/cm^2)
θ_{syn}	0 (mV)
α	12 (msec^{-1})
β	0.1 (msec^{-1})
C_m	1.0 (μF/cm^2)
threshold(spike)	-55 (mV)

Table S2: Parameters for Traub neuron model.

parameter	value
g_L	0.2 (mS/cm^2)
g_{Na}	100 (mS/cm^2)
g_K	80 (mS/cm^2)
E_L	-67 (mV)
E_{Na}	50 (mV)
E_K	-100 (mV)
g_{syn}	0.01 (mS/cm^2)
α	12 (msec^{-1})
β	0.5 (msec^{-1})
C_m	1.0 (μF/cm^2)
V_{res}	-95 (mV)
threshold(spike)	-55 (mV)

n. Therefore, decreasing ϕ reduces the firing rate by producing a deep afterhyperpolarization. The difference between the lowest value of the voltage and the firing threshold is about 15 mV at $\phi = 5$. This is in agreement with experimental results for fast-firing inhibitory interneurons. However, for $\phi = 9$ this difference disappears and the spikes can not be distinguished (see **figure S7**).

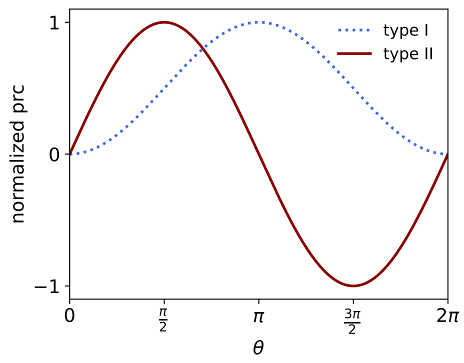

Figure S1: **Phase response curves of the type I and type II phase oscillators.**

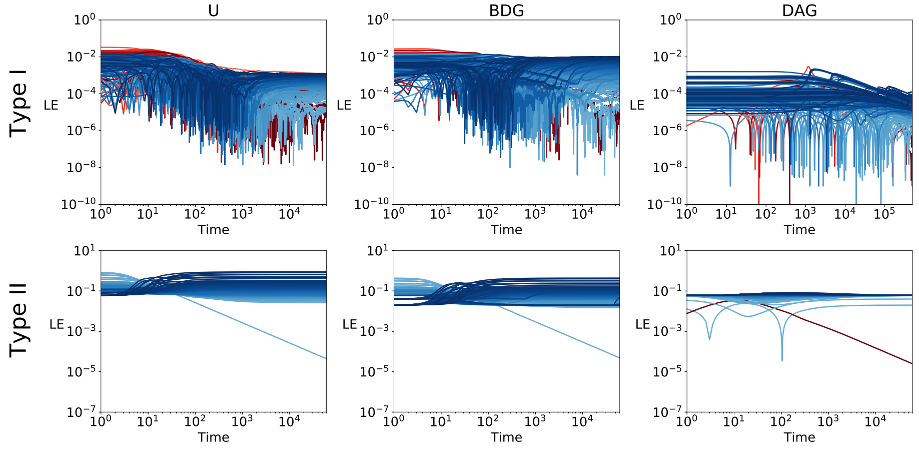

Figure S2: **The Lyapunov exponents of the extended Kuramoto model on different network structures.** The Lyapunov exponents versus time (log-log scale) for identical (top) type I and (bottom) type II excitatory phase oscillators connected by different scale-free networks. The sign of the Lyapunov exponents are colour coded, where blue and red indicate negative and positive values, respectively.

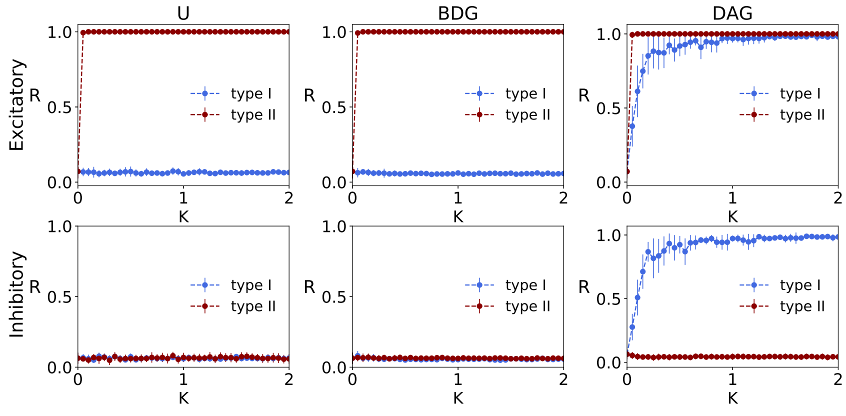

Figure S3: **Synchronization of the excitatory and inhibitory phase oscillators.** The stationary order parameters versus the coupling strengths for the scale-free undirected and oriented networks with (top) excitatory and (bottom) inhibitory phase oscillators.

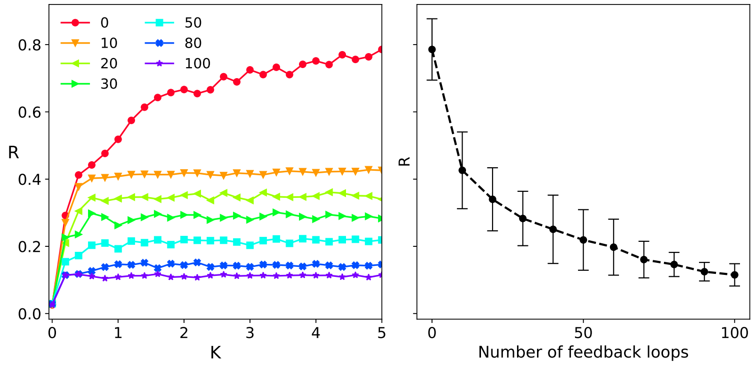

Figure S4: **Comparison of synchronization of type I phase oscillators on the scale-free directed networks with different numbers of feedback loops.** The stationary order parameter versus (left) the coupling strength and (right) the number of feedback loops (for $K = 5$). The networks have $N = 1000$ nodes and $\gamma = 3$. The results are averaged over 30 realizations.

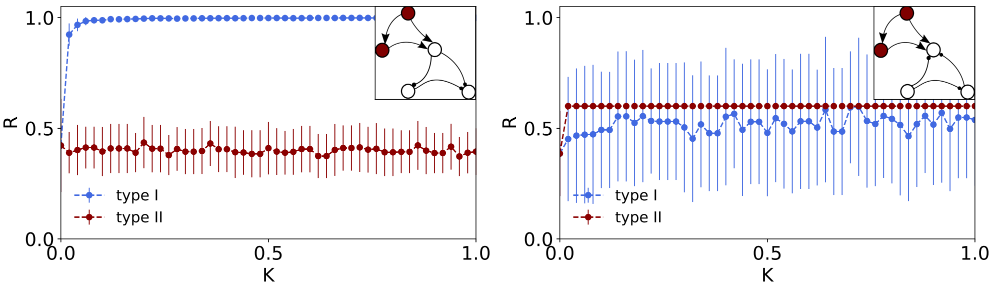

Figure S5: **Synchronization of the networks constructed from inhibitory and excitatory feedforward motifs.** The stationary order parameters for type I and type II phase oscillators versus the coupling strengths for (left) connected and (right) disconnected networks. The excitatory and inhibitory oscillators are distinguished by red and white colours. The results are averaged over 30 realizations.

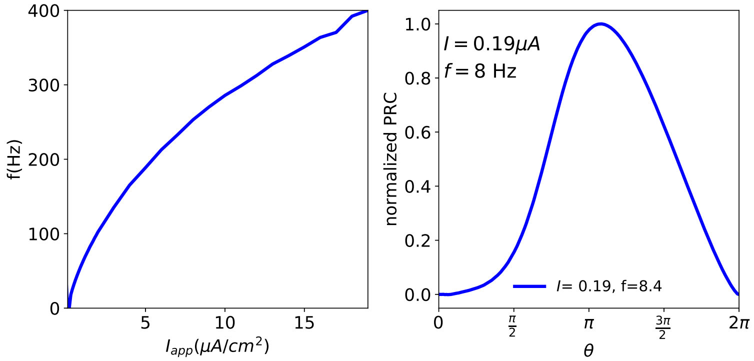

Figure S6: **Dynamical properties of a type I Wang-Buzsáki inhibitory neuron.** (Left) The firing frequency versus applied current. (Right) The phase response curve (PRC) of the neuron.

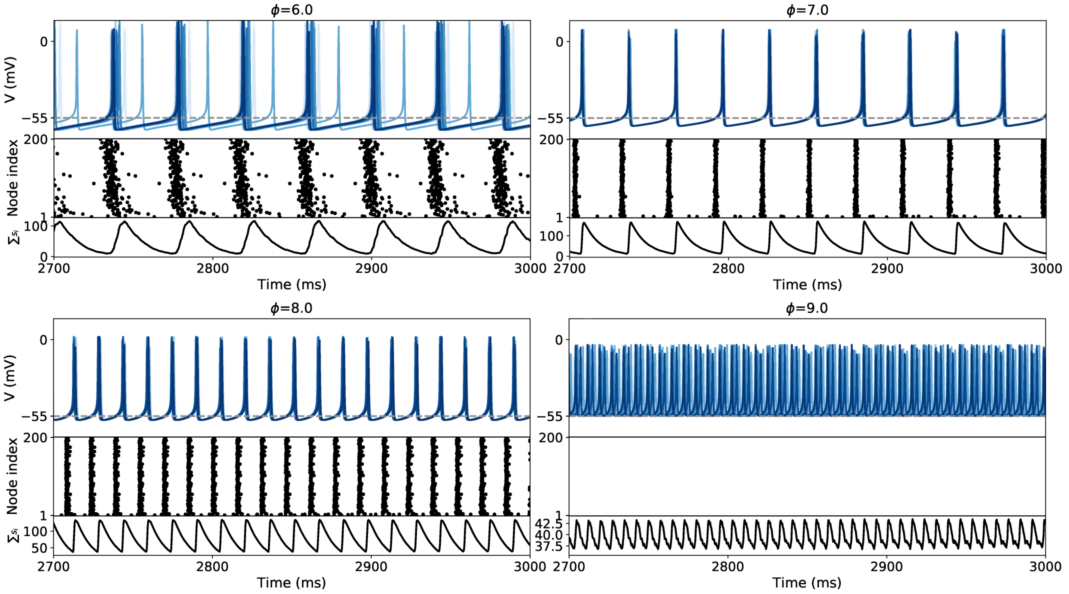

Figure S7: **Effects of changing ϕ on after-hyperpolarization and synchronization of Wang-Buzsáki neurons.** From top to bottom, each panel includes the membrane potential of the neurons versus time, the raster plot, and the sum of the synaptic gates $\sum_i s_i$ versus time for an specified ϕ. The networks are scale-free DAGs with $N = 200$ nodes and $\gamma = 3$. The coupling constant is $K = 1.25$.

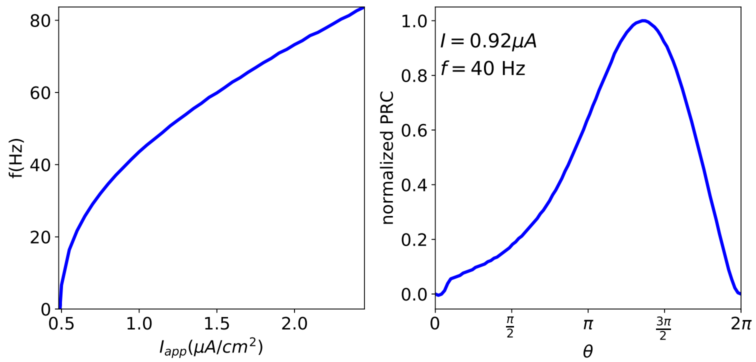

Figure S8: **Dynamical properties of a type I Traub excitatory neuron.** (Left) The firing frequency versus applied current. (Right) The phase response curve (PRC) of the neuron.

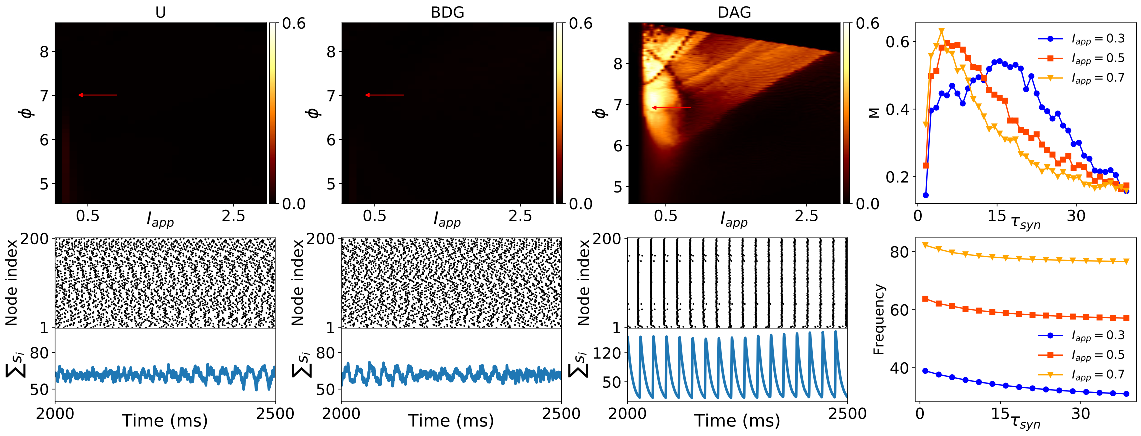

Figure S9: **Synchronization of Wang-Buzsáki inhibitory neurons coupled via scale-free undirected and oriented graphs.** (First three left columns) The top panel displays the voltage synchrony of the networks on $I_{app} - \phi$ phase space. The bottom panel represents the raster plots and the sum of the synaptic gates $\sum_i s_i$ for three network instances using the parameters specified by the arrows at the top plots. The synapse parameters are $\alpha = 12ms^{-1}, \beta = 0.1ms^{-1}, g_{syn} = 0.1mS/cm^2$. (Right column) The top and bottom panels show the effects of synaptic time constant ($\tau_{syn} = 1/\beta$) on synchrony and frequency of oscillation for different values of applied currents, respectively at $K = 1.25$. Structural parameters of the networks are valued as $N = 200$, and $\gamma = 3$.

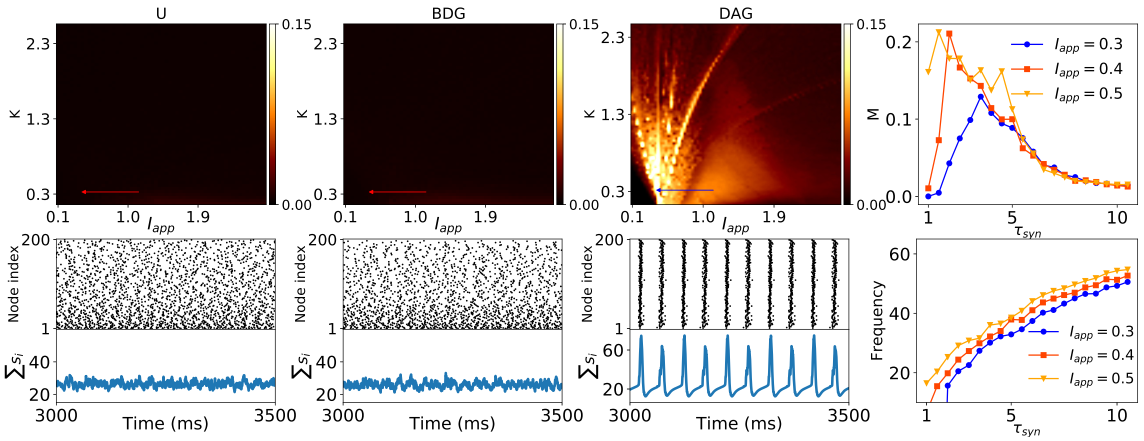

Figure S10: **Synchronization of Traub excitatory neurons coupled via scale-free undirected and oriented graphs.** (First three left columns) The top panel shows the voltage synchrony of the networks on $I_{app} - K$ phase space. The bottom panel shows the raster plots and the sum of the synaptic gates $\sum_i s_i$ for three network instances using the parameters specified by the arrows at the top plots. The synapse parameters are $\alpha = 12ms^{-1}, \beta = 0.5ms^{-1}, g_{syn} = 0.01mS/cm^2$. (Right column) The top and bottom panels represent the effects of synaptic time constant ($\tau_{syn} = 1/\beta$) on spike synchrony and frequency of oscillation, respectively at $K = 0.3$. Structural parameters of the network are valued as $N = 200$, and $\gamma = 3$.

References

[1] Alan Wolf, Jack B Swift, Harry L Swinney, and John A Vastano. Determining lyapunov exponents from a time series. *Physica D: Nonlinear Phenomena*, 16(3):285–317, 1985.

[2] Marek Balcerzak and Danylo Pikunov. The fastest, simplified method of estimation of the largest lyapunov exponent for continuous dynamical systems with time delay. *Mechanics and Mechanical Engineering*, 21(4):985–994, 2017.
